# The Hydrogen Sulfide Donor NaHS Delays Programmed Cell Death in Barley Aleurone Layers by Acting as an Antioxidant

**DOI:** 10.1155/2015/714756

**Published:** 2015-05-11

**Authors:** Ying-Xin Zhang, Kang-Di Hu, Kai Lv, Yan-Hong Li, Lan-Ying Hu, Xi-Qi Zhang, Long Ruan, Yong-Sheng Liu, Hua Zhang

**Affiliations:** ^1^School of Biotechnology and Food Engineering, Hefei University of Technology, Hefei 230009, China; ^2^Anhui Academy of Agricultural Sciences, Hefei 230031, China

## Abstract

H_2_S is a signaling molecule in plants and animals. Here we investigated the effects of H_2_S on programmed cell death (PCD) in barley (*Hordeum vulgare* L.) aleurone layers. The H_2_S donor NaHS significantly delayed PCD in aleurone layers isolated from imbibed embryoless barley grain. NaHS at 0.25 mM effectively reduced the accumulation of superoxide anion (·O_2_^−^), hydrogen peroxide (H_2_O_2_), and malondialdehyde (MDA), promoted the activity of superoxide dismutase (SOD), guaiacol peroxidase (POD), catalase (CAT), and ascorbate peroxidase (APX), and decreased those of lipoxygenase (LOX) in isolated aleurone layers. Quantitative-PCR showed that NaHS treatment of aleurone tissue led to enhanced transcript levels of the antioxidant genes *HvSOD1, HvAPX, HvCAT1*, and *HvCAT2* and repressed transcript levels of *HvLOX* (lipoxygenase gene) and of two cysteine protease genes *HvEPA* and *HvCP3-31*. NaHS treatment in gibberellic acid- (GA-) treated aleurone layers also delayed the PCD process, reduced the content of ·O_2_^−^, and increased POD activity while decreasing LOX activity. Furthermore, *α*-amylase secretion in barley aleurone layers was enhanced by NaHS treatment regardless of the presence or absence of GA. These data imply that H_2_S acted as an antioxidant in delaying PCD and enhances *α*-amylase secretion regardless of the presence of GA in barley aleurone layers.

## 1. Introduction

Programmed cell death (PCD), a response of plants to biotic and abiotic stresses, can also occur during the normal course of development [1]. Cereal aleurone layers undergo gibberellic acid- (GA-) stimulated PCD process following germination and therefore provide a convenient model for studying PCD [2]. PCD in barley aleurone layers occurs only after cells become highly vacuolated and is accompanied with loss of plasma membrane integrity and increased cysteine protease activity [3]. However, the hallmarks of apoptosis in animal cells, including internucleosomal DNA ladders and formation of apoptotic bodies, are not observed in aleurone cells [4]. Reactive oxygen species (ROS) such as superoxide anion (·O_2_^−^), hydrogen peroxide (H_2_O_2_), and hydroxyl radicals are key players in the PCD process in both plants and animals [1, 4, 5]. For instance, intracellular H_2_O_2_ overproduction or exogenous H_2_O_2_ application results in a rapid death in GA-treated aleurone protoplasts [6]. Antioxidant enzymes such as catalase (CAT), guaiacol peroxidase (POD), ascorbate peroxidase (APX), and superoxide dismutase (SOD) are responsible for ROS scavenging, thereby keeping homeostatic levels of ROS. PCD in plants is also accompanied by increased protease activity [2, 7]. For instance, cysteine protease activation is instrumental in PCD of soybean cells, while ectopic expression of cystatin, a cysteine protease inhibitor gene, inhibits cysteine protease activity and blocks PCD [7]. What is more, the increase in cysteine protease and aspartic protease activities is also observed in GA-treated barley aleurone layers [2].

Hydrogen sulfide (H_2_S), similar to nitric oxide (NO) and carbon monoxide (CO), is an important endogenous gaseous signaling molecule in animal cells [8]. Accumulating evidence shows that H_2_S is involved in various processes in plants, such as response to pathogen attack, seed germination, root organogenesis, abiotic stress tolerance, guard cell movement, and postharvest senescence of fruits and vegetables [9–17]. In particular, during abiotic stresses and postharvest storage, H_2_S acts as an antioxidant to counteract excessive ROS to promote seed germination and alleviate postharvest senescence [10, 15, 16]. More recently, H_2_S is found to delay PCD in GA-treated wheat aleurone layers by modulation of glutathione (GSH) and heme oxygenase 1 expression [18]. However, whether H_2_S has a role in regulating PCD in barley aleurone layers treated with GA or not and whether ROS and ROS-scavenging enzymes participate in the role of H_2_S are still unknown. In the present research, we find that the H_2_S donor NaHS effectively delays PCD in barley aleurone layers regardless of the presence of GA, through the enhancement in antioxidant enzyme genes expression and antioxidant enzyme activity and decrease in protease gene expression.

## 2. Materials and Methods

### 2.1. Materials and Treatments

Grains of barley (*Hordeum vulgare* L.) were kindly supplied by Jiangsu Academy of Agricultural Sciences, Jiangsu Province, China. Grains were surface-sterilized as described by Chrispeels and Varner [19]. In brief, embryo end of the grain was removed, and fifteen half-grains were imbibed in distilled water at 25°C for 3 d on Petri dishes and further used for NaHS or gibberellic acid (GA) plus CaCl_2_ treatment. H_2_S donor NaHS and GA were purchased from Sigma.

### 2.2. Cell Viability Analysis in Barley Aleurone Layers

Barley half-grains pretreated with water for 3 d were incubated in different concentrations of NaHS (0, 0.005, 0.025, 0.05, 0.25, or 0.5 mM) or 0.25 mM NaHS + 5 *μ*M GA (in 10 mM CaCl_2_) at 25°C for 5 d prior to isolation of aleurone layers from half-grains. To determine the number of dead cells, three aleurone layers per treatment were stained with 0.4% trypan blue [20] for 10 min and observed with Nikon Eclipse 80 i fluorescence microscope (Nikon, Japan). The percentage of dead cells was determined by the calculation of blue or purple cells compared to the total number of cells in randomly selected fields from three different aleurone layers per treatment.

### 2.3. Detection of Reactive Oxygen Species Using Fluorescent Probe

A reactive oxygen species kit 2′,7′-dichlorodifluorescein diacetate (DCHF-DA) (Cayman Chemical, America) which is a fluorogenic probe in living cells was used to detect ROS content [21]. Three aleurone layers per treatment were rinsed with water three times and incubated with 5 *μ*M DCHF-DA for 20 min at 37°C in the dark according to manufacturer's instructions. The fluorescence of dichlorofluorescein DCF (excitation at 488 nm, emission at 525 nm) was observed using a Nikon Eclipse 80 i fluorescence microscope (Nikon, Japan). Nonstained aleurone layers were used as negative control. The experiment was repeated three times and similar results were obtained.

### 2.4. Determination of the Contents of Superoxide Anion, Hydrogen Peroxide, and Malondialdehyde

·O_2_^−^, H_2_O_2_, and MDA contents were measured according to the method in [22]. Embryoless half-grains were pretreated with sterile water for 3 d and incubated in sterile H_2_O, 0.25 mM NaHS, and 0.25 mM NaHS + 5 *μ*M GA. Three independent experiments with three replicates of 15 half-grains (0.45 ± 0.001 g) were sampled for each treatment every 24 h until the fifth day.

### 2.5. Assays of the Activity of Antioxidant Enzymes and Lipoxygenase

Activity of SOD (EC 1.15.1.1), CAT (EC 1.11.1.6), APX (EC 1.11.1.11), and POD (EC 1.11.1.7) was determined according to García-Limones et al. [23] and that of LOX (EC 1.13.11.12) followed the description by Surrey [24]. Frozen grain samples (0.45 ± 0.001 g) were homogenized with 1 mL of 200 mM ice-cold phosphate buffer (pH 7.8) containing 1.0 mM ethylenediaminetetraacetic acid (EDTA). The homogenate was centrifuged at 12,000 g at 4°C for 20 min, and the supernatant was used for activity measurement.

For LOX, three independent replicates of 15 half-grains (0.45 ± 0.001 g) in three independent experiments per treatment were homogenized with 1 mL of 200 mM phosphate buffer (pH 6.0). The homogenate was centrifuged at 15,000 g at 4°C for 10 min, and the supernatant was used for the enzyme assay. The assay mixture in a total volume of 3 mL contained 200 mM borate buffer (pH 6.0), 0.25% linoleic acid, 0.25% tween-20, and 50 *μ*L of enzyme extract. The reaction was carried out at 25°C for 5 min, and the activity of LOX was determined in the presence of linoleic acid by monitoring the changes in absorbance at 234 nm.

### 2.6. Quantitative PCR Analysis

Total RNA was isolated from five aleurone layers using the plant RNeasy kit (Forgene, China) according to the manufacturer's instructions. Total RNA (500 ng) from different treatments was used for first-strand cDNA synthesis in a 20 *μ*L reaction volume containing 4 *μ*L 5 × PrimeScript RT Master Mix (TaKaRa). Quantitative PCR was performed using a StepOnePlus Real-Time PCR System (Applied Biosystems, Foster City, CA, USA) with SYBR Premix Ex Taq (TaKaRa Bio Inc, China) according to the manufacturer's instructions. cDNA was amplified by PCR using the following primers:* HvActin* (accession number: LOC548170) forward (5′-TCTCACGGACTCCCTTT-3′) and* HvActin* reverse (5′-CACTGAGCACGATGTTTC-3′);* HvCAT1* (accession number: HVU20777) forward (5′-AAG­ACC­GTT­TCC­TCC­AGC-3′) and reverse (5′ATT­CAA­GGC­TAC­CGC­ACA-3′);* HvCAT2* (accession number: HVU20778) forward (5′-CGC­CTT­CAA­GCC­CAA­CCC­A-3′) and reverse (5′-TTC­TCC­CTC­TTT­CCA­ACC­AC-3′);* HvSOD1* (accession number: JQ364454) forward (5′-CGA­TAG­CCA­GAT­TCC­TTT­G-3′) and reverse (5′-TCC­ACC­AGC­ATT­TCC­AGT­A-3′);* HvAPX* (accession number: AJ006358) forward (5′-CTA­CTA­CTG­CTG­CTA­CTA­TGC­G-3′) and reverse (5′-CAC­TGA­CAG­CGT­TCA­AGG­TAT-3′);* HvLOX* (accession number: AJ966349) forward (5′-CCG­CTC­TGA­CCC­ATT­TCG-3′) and reverse (5′-TGC­TCC­TTG­ACC­TCC­ACC­TT-3′);* HvICY* (accession number: AJ536590) forward (5′-TCG­TCG­TGC­CGT­TTA­CTC-3′) and reverse (5′-TTG­GCC­TTC­TTG­TTG­TGC-3′);* HvEPA* (accession number: HVU94591) forward (5′-CCC­GTG­TCG­GTG­GCA­ATA-3′) and reverse (5′-GCA­TCC­TGA­TGT­AAC­CCT­TCT­C-3′);* HvCP3-31* (accession number: AB377533) forward (5′-ACA­ACC­TCC­GCT­ACA­TCG-3′) and reverse (5′-CCC­TTC­TTC­CTC­CAG­TCG-3′). Relative gene expression was presented as values relative to control* HvActin* transcript level, after normalization to the control* HvActin* transcript levels.

### 2.7. Assays of Secreted *α*-Amylase Activity

Embryoless barley half-grains were incubated in distilled water for 3 d and then treated with various concentrations of NaHS in presence or absence of 20 *μ*M GA and 10 mM CaCl_2_.

Agar-starch medium (containing 4% agar and 0.1% starch) was used to detect *α*-amylase activity secreted by aleurone layers in NaHS treatment without GA and CaCl_2_ for 24 h. Aleurone layers which were prepared as described above were placed on agar-starch medium for 16 h after which the agar-starch was stained with 0.6% I_2_ and 6% KI solution to show digested starch zones. The experiment was repeated three times and similar results were obtained.

Twenty embryoless half-grains were imbibed in distilled water at 25°C for 3 d on Petri dishes and incubated in Erlenmeyer flasks which contained different concentrations of NaHS in 20 *μ*M GA and 10 mM CaCl_2_. Incubation medium was sampled after 24 h and heated at 70°C for 15 min to eliminate *β*-amylase activity. Amylase secreted to the medium was visualized in 10% native PAGE gels by the starch-iodine method according to [25]. To visualize *α*-amylase activity, the gel was incubated at 25°C for 30 min in 50 mM PBS (pH 7.0) containing 1% boiled soluble starch. After being washed three times with distilled water, the gel was stained with 0.6% I_2_ and 6% KI solution. The experiment was repeated three times and similar results were obtained.

The DNS method for the determination of secreted *α*-amylase activity in medium was performed in 0.01 M sodium acetate buffer, pH 5.4. The reaction mixture containing 1% soluble starch was incubated at 25°C for 5 min without substrate. Then, the reaction was initiated by adding the substrate and was continued for an additional 10 min at 37°C. The reaction was terminated and hydrolysis was determined with 3,5-dinitrosalicylic acid reagent as modified by Noelting and Bernfeld [26].

### 2.8. Statistical Analysis

Statistical significance in all experiments was tested by one-way analysis of variance (ANOVA), and the results are expressed as the mean values ± standard deviation (SD) of three independent experiments with three replicates for each. Fisher's least significant differences (LSD) were calculated following a significant (*P* < 0.01 or *P* < 0.05) *t*-test.

## 3. Results

### 3.1. Programmed Cell Death in Barley Aleurone Layers Is Delayed by the H_2_S Donor NaHS

**Figure 1:**
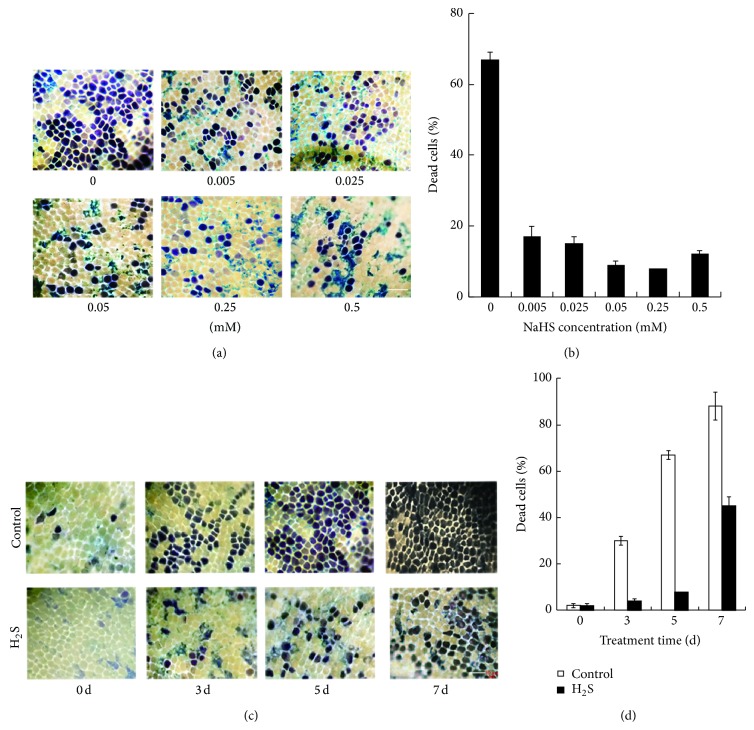
Effect of H_2_S donor NaHS on cell viability in barley aleurone layers. ((a), (b)) Aleurone layers are incubated in different concentrations of NaHS (0, 0.005, 0.025, 0.05, 0.25, and 0.5 mM) for 5 d at 25°C. After staining with trypan blue, the images are obtained by light microscopy with blue or purple indicating dead cells. ((c), (d)) Time course of PCD in barley aleurone layers treated with NaHS (H_2_S) or water (Control). Aleurone layers are incubated in 0.25 mM NaHS or water for 0, 1, 3, 5, and 7 d at 25°C and are stained with trypan blue. Digital images of barley aleurone layers ((a), (c)) and percentages of dead cells ((b), (d)) are shown. Bar, 50 *μ*m. Data are means ± SD of three different aleurone layers per treatment.

To test the effect of H_2_S on the PCD process, water-pretreated barley half-grains were incubated in different concentrations of NaHS for 5 days. Aleurone layers are isolated from half-grains and stained with trypan blue to visualize dead cells. NaHS treatments ranging from 0.005 to 0.5 mM significantly decrease cell death compared with water controls (Figures 1(a) and 1(b)). Only 9% of cells die in aleurone layers treated with 0.25 mM NaHS while approximately 67% of cells of aleurone layers incubated in water undergo PCD. As shown in Figure 1, NaHS at 0.25 mM is most effective in delaying PCD in barley aleurone layers, and this concentration is used in subsequent experiments.

A time course of cell death in aleurone layers treated with 0.25 mM NaHS is shown in Figures 1(c) and 1(d). After 7 days incubation in water, about 90% aleurone cells are dead in contrast to only 45% cell death in NaHS-treated layers. Together, these findings show that barley aleurone cells undergo PCD naturally in the absence of GA and that the H_2_S donor NaHS effectively delays the PCD process.

### 3.2. NaHS Treatment Reduces the Accumulation of Reactive Oxygen Species in Non-GA-Treated Barley Aleurone Layers

**Figure 2:**
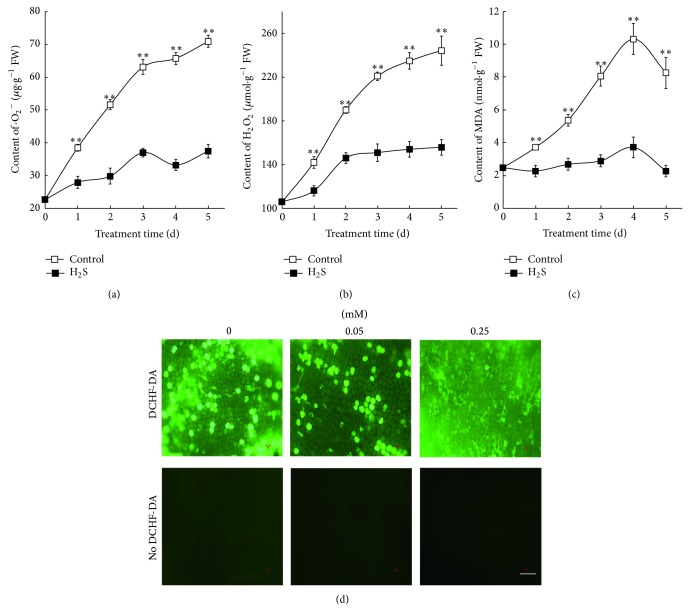
Effects of NaHS on the contents of ·O_2_^−^ (a), H_2_O_2_ (b), and MDA (c) in barley aleurone layers. Aleurone layers treated with 0, 0.05, and 0.25 mM NaHS for 1 d are incubated DCHF-DA and are observed by fluorescence microscopy (d). Bar, 100 *μ*m. Data are expressed as means ± SD of three independent experiments with three replicates of 15 grains per treatment. The symbols ∗ and ∗∗ mean significant difference at *P* < 0.05 and *P* < 0.01 between NaHS (H_2_S) and water (control) treatment, respectively.

Because ROS are tightly associated with the promotion of PCD in barley aleurone cells [6], we examine the contents of ·O_2_^−^, H_2_O_2_, and MDA in non-GA-treated barley aleurone layers in the presence and absence of NaHS. As shown in Figure 2(a), ·O_2_^−^ content in control barley aleurone layers accumulates rapidly during the 5 days of incubation. However, ·O_2_^−^ content in NaHS-treated layers accumulates slowly until day 3 and keeps stable on day 5.

The assay of H_2_O_2_ shows that layers incubated in NaHS produce less H_2_O_2_ than those incubated in water (Figure 2(b)). H_2_O_2_ content increases rapidly in control aleurone layers during the whole incubating time, whereas a slower increase in H_2_O_2_ content was observed in NaHS treatment on the first two days followed by a plateau.

MDA is determined as an index of lipid peroxidation. As shown in Figure 2(c), MDA content increases rapidly in water controls until day 4 followed by a decrease. In contrast, NaHS treatment significantly lowers the level of MDA (Figure 2(c)).

We use the ROS-sensitive fluorescent probe DCHF-DA to visualize the production of ROS in barley aleurone layers (Figure 2(d)). Fluorescence from layers which are incubated in 0.05 and 0.25 mM NaHS is much less intense than water controls. More weak fluorescence is detected in tissue incubated in 0.25 mM NaHS.

### 3.3. Effects of NaHS on Antioxidant Enzymes and Lipoxygenase in Non-GA-Treated Barley Aleurone Layers

**Figure 3:**
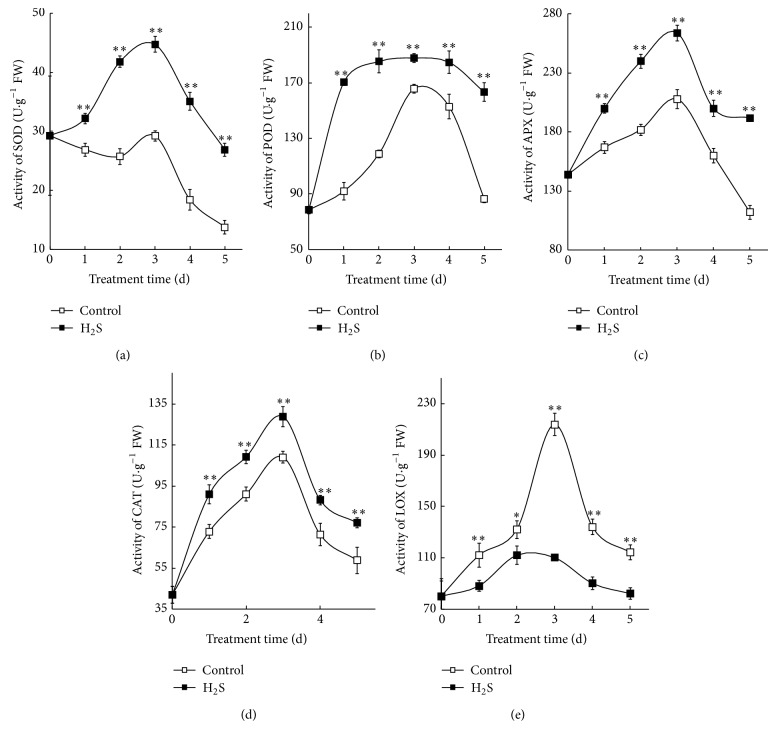
Effects of NaHS on the activity of SOD (a), POD (b), APX (c), CAT (d), and LOX (e) in barley aleurone layers. Data are expressed as means ± SD of three independent experiments with three replicates of 15 grains per treatment. The symbols ∗ and ∗∗ mean significant difference at *P* < 0.05 and *P* < 0.01 between control and T, respectively.

We examine the activity of the ROS metabolizing enzymes SOD, POD, CAT, APX, and LOX in barley aleurone layers that are incubated in 0.25 mM NaHS and water (Figure 3). The activity of SOD increases to maximum on day 3 and then declines in NaHS-treated layers. In contrast, SOD activity in water controls fluctuates slightly up to day 3 followed by a significant decrease (Figure 3(a)).

Figure 3(b) shows changes in POD activity in NaHS-treated and water control layers. NaHS significantly increases POD activity on day 1 and remains high until day 4. In comparison, POD activity in water controls increases gradually and peaks on day 3 followed by a sharp decline. NaHS treatment maintains significantly higher levels of POD activity compared with water control during the whole treatment time. APX activity increases during the first 3 days of incubation and peaks on day 3 followed by a decrease in both NaHS-treated and water controls. However, APX activity in NaHS treatment is always significantly higher than that of control (Figure 3(c)).

Changes in CAT activity are shown in Figure 3(d). In both NaHS and water controls, CAT activity increases gradually up to day 3 and then decreases sharply. However, CAT activity from NaHS-treated layers is always significantly higher than those in control layers.

Figure 3(e) shows the changes in LOX activity in barley aleurone layers. LOX activity in water control increases dramatically and peaks on day 3 followed by a decrease. In contrast, NaHS treatment significantly decreases LOX activity, being about 50% of that of water control on day 3.

### 3.4. Transcript Analysis of* HvSOD1*,* HvCAT1*,* HvCAT2*,* HvLOX*, Cysteine Protease (*HvCP3-31* and* HvEPA*), and Cystatin (*HvICY*) in Non-GA-Treated Barley Aleurone Layers

**Figure 4:**
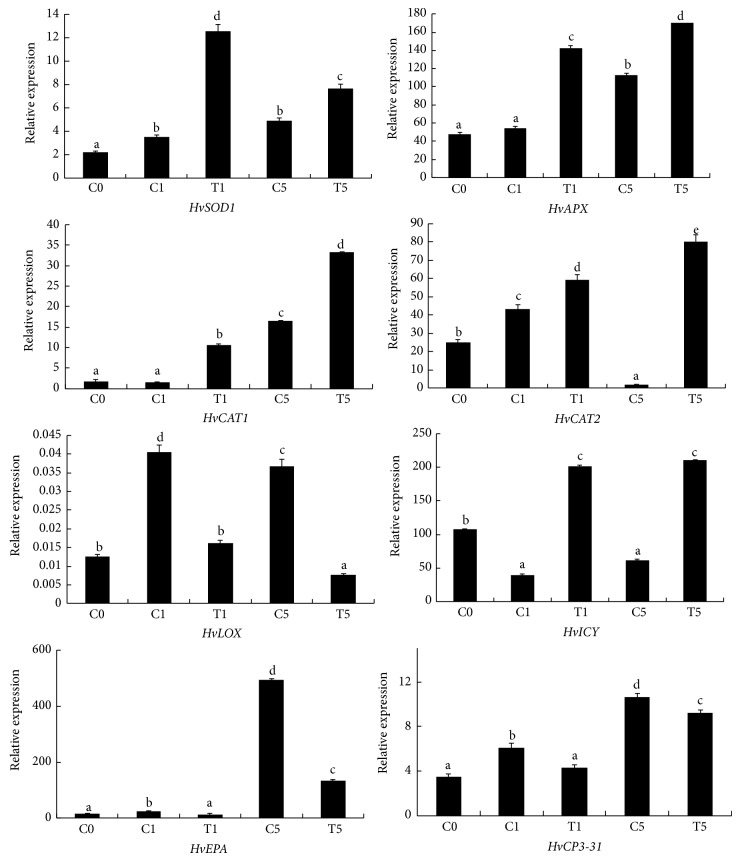
Effects of H_2_S on the expression of* HvSOD1*,* HvCAT1*,* HvCAT2*,* HvLOX*, cystatin (*HvICY*), and the cysteine proteases* HvCP3-31* and* HvEPA* in barley aleurone layers. Aleurone layers are incubated in NaHS (T) or water (C) and total RNA is obtained at 0, 1, and 5 d. Means and SD values are calculated from three independent experiments. Within each identified gene, bars with different letters are significantly different in comparison with the corresponding control at *P* < 0.01 according to Fisher's least significant differences (LSD).

We examine the expression of* HvSOD1*,* HvCAT1*,* HvCAT2*,* HvLOX*, the cysteine proteases* HvCP3-31* and* HvEPA,* and cystatin (*HvICY*) in NaHS-treated barley aleurone layers and water controls (Figure 4). Compared with water controls, NaHS induces higher expression of* HvSOD1*,* HvAPX*,* HvCAT1,* and* HvCAT2* on days 1 and 5.* HvLOX* expression increases in water control layers on days 1 and 5 compared with day 0, while NaHS treatment sustains lower transcript of* HvLOX* than water control, especially on day 5. PCD in barley aleurone layers is accompanied with increased cysteine protease activity [3]. Accordingly, we determine the expression of the cysteine proteinases* HvEPA* and* HvCP3-31* and the cystatin* HvICY* in NaHS treatment and water control. The expression of* HvEPA* and* HvCP3-31* increases in water controls on days 1 and 5, whereas their expression is much lower in NaHS-treated tissue. The expression of* HvICY* was enhanced in NaHS-treated layers, whereas less transcript of* HvICY* is observed in water controls.

### 3.5. NaHS Delays PCD in GA-Treated Barley Aleurone Layers

**Figure 5:**
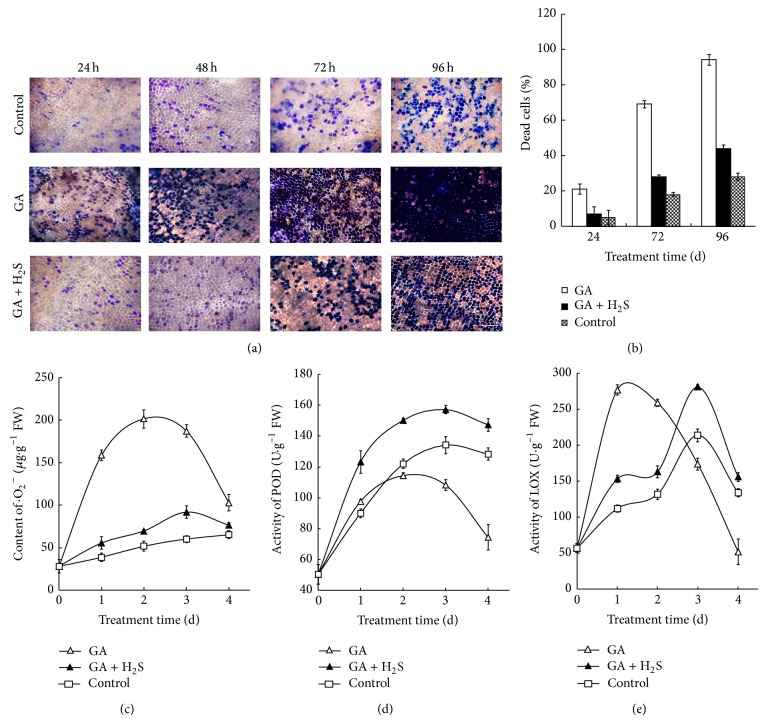
NaHS delays PCD in GA-treated barley aleurone layers. Aleurone layers are incubated in water, GA, or GA + NaHS (GA + H_2_S) and, after being stained with trypan blue, images are obtained by light microscopy (a) and the percentage of dead cells is shown in (b). Content of ·O_2_^−^ (c), activity of POD (d), and LOX (e) are measured on 0, 1, 2, 3, and 4 d. Bar, 100 *μ*m. Data in (c) and (d) are expressed as means ± SD of three independent experiments with three replicates of 15 grains per treatment.

PCD in barley aleurone layers is tightly regulated by GA and abscisic acid (ABA). We therefore assess whether H_2_S can ameliorate PCD in GA-treated barley aleurone layers. As shown in Figure 5(a), the accumulation of dead cells increases rapidly from 24 to 96 h in GA-treated barley aleurone layers, whereas 0.25 mM NaHS treatment significantly delays the rate of PCD. After incubation for 96 h, about 90% cells in GA-treated layers are dead, while half of cells are still alive in NaHS-treated layers (Figure 5(b)). In water control, much less cells undergo PCD compared with the counterpart of GA and GA plus NaHS.

Determination of ·O_2_^−^ content shows that NaHS treatment maintains lower levels of ·O_2_^−^ in GA-treated barley aleurone layers (Figure 5(c)). After a rapid increase during the first 2 days of incubation, the content of ·O_2_^−^ in GA-treated layers decreases till day 4. In contrast, ·O_2_^−^ content in NaHS plus GA treatment increases more slowly until day 3. A comparable but lower ·O_2_^−^ content was observed in water control compared with NaHS plus GA treatment.

Figure 5(d) shows the effect of H_2_S on POD activity in GA-treated barley aleurone layers. In both NaHS treatment and GA control, POD activity in GA-treated barley aleurone layers increases gradually up to day 3 and day 2, respectively, and decreases thereafter. However, the activity of POD in NaHS-treated aleurone layers is always significantly higher than those in water controls and GA treatment alone.

Figure 5(e) shows that NaHS treatment maintains lower levels of LOX activity compared with water control during the first 2 days of GA treatment. LOX activity in GA treatment increases dramatically on day 1 followed by a gradual decrease, while, in NaHS-treated tissue, the activity increases more slowly till day 3 followed by a decline. After day 3, LOX activity in NaHS plus GA is higher than that in GA treatment.

### 3.6. H_2_S Donor Promotes *α*-Amylase Secretion in Barley Aleurone Layers Regardless of GA

**Figure 6:**
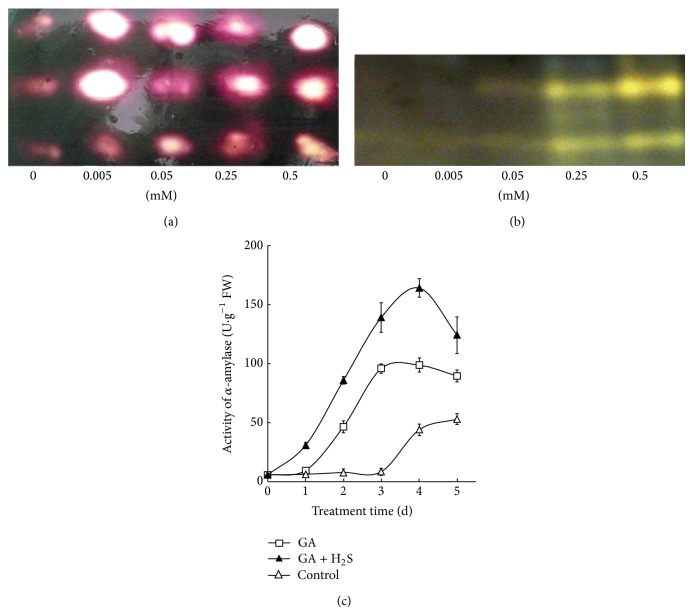
NaHS increases the activity of *α*-amylase in incubation medium of barley aleurone layers in the absence (a) and presence ((b), (c)) of GA. Barley aleurone layers isolated from treated embryoless half grains are incubated in 0, 0.005, 0.05, 0.25, or 0.5 mM NaHS for 24 h ((a), (b)). After incubation, aleurone layers are placed on agar-starch medium (containing 4% agar and 0.1% starch) for 16 h. Agar plates are stained with I_2_-KI solution to detect the activity of *α*-amylase (a). (b) shows native PAGE analysis of *α*-amylase activity in incubation medium surrounding the aleurone layers. (c) indicates secreted *α*-amylase activity in incubation medium surrounding barley aleurone layers treated with water (control), GA, or GA + NaHS (GA + H_2_S) at different times of incubation. Data in (c) are expressed as means ± SD of three independent experiments with three replicates of 20 embryoless half grains per treatment.

Secretion of *α*-amylase is a characteristic response of aleurone cells to GA. We therefore test whether the ameliorating effect of H_2_S on PCD affects the release of *α*-amylase. As shown in Figure 6(a), NaHS promotes *α*-amylase release in water-treated aleurone layers. In the presence of GA, NaHS treatment also enhances *α*-amylase release from barley aleurone layers (Figure 6(b)) with 0.5 mM NaHS exhibiting optimal effect.  Figure 6(c) shows the time changes in *α*-amylase secretion in water control, GA-treated aleurone layers, and GA plus 0.25 mM NaHS treatment. The accumulation of *α*-amylase in incubation medium in GA-treated layers increases and peaks on day 3 followed by a plateau but addition of 0.25 mM NaHS brings about a more rapid increase till day 4 followed by a decrease on day 5 (Figure 6(c)). The activity of *α*-amylase released following GA + NaHS treatment is significantly higher than that of layers incubated only in GA during the whole treatment time. As expected, much lower *α*-amylase activity was only observed in water control after 3 days of incubation. Together, this result indicates that H_2_S delays PCD in barley aleurone layers and meanwhile promotes *α*-amylase release regardless of the presence of GA.

## 4. Discussion

H_2_S participates in multiple processes in plants [27]. In this paper, we show that H_2_S delays PCD in barley aleurone layers regardless of the presence or absence of GA. In the absence of GA, PCD in barley aleurone layers is evident on day 3 of incubation, while H_2_S delays PCD at an optimal concentration of 0.25 mM (Figure 1). Consistent with the reports that GA accelerates PCD process in barley aleurone layers, GA treatment triggers cell death in about 90% aleurone cells at 72 h (Figure 5(a)). GA-induced cell death is slowed by the addition of NaHS and this H_2_S donor also prolongs the phase of *α*-amylase production in GA-treated layers. The promoting effect of NaHS on *α*-amylase synthesis and its prolongation of cell survival indicate that 2.5 mM NaHS does not affect aleurone cell function.

ROS, such as ·O_2_^−^ and H_2_O_2_, are inducers of PCD in plant and animal cells [1]. It is reported that the peroxidation of membrane lipids and damage to the plasma membrane can occur when the rate of ROS production overcomes the cells' ability for scavenging ROS [27]. Overproduction of ROS and oxidative damage are universal events in PCD in plant cells [28]. In this paper, we show that the content of ·O_2_^−^ increases in parallel with cell death in GA-treated layers (Figure 5(c)). In non-GA-treated layers, the burst of ·O_2_^−^ and H_2_O_2_ and the accumulation of MDA are also accompanied by PCD (Figure 2). These results suggest that ROS play a key role both in GA-treated [29] and in non-GA-treated aleurone layers (Figures 2(a), 2(b), and 5(c)).

Aleurone cells contain a suite of ROS-metabolizing enzymes. GA-induced PCD in layers is accompanied by a decline in activity of ROS metabolizing enzymes which leads to increased susceptibility of aleurone cells to ROS [29]. A novel aspect of our work is that NaHS treatment effectively reduces the accumulation of ROS in barley aleurone layers regardless the presence of GA (Figures 2 and 5), thereby delaying PCD process in these cells. We propose that the H_2_S donor reduces ROS accumulation in layers by increasing the activity of ROS-scavenging enzymes. The data in present study show that H_2_S treatment maintains significantly higher POD activity in GA-treated layers (Figure 5(d)) and higher SOD, POD, CAT, and APX activity in non-GA-treated layers (Figure 3). The increased activity of ROS-scavenging enzymes in NaHS-treated likely promotes the cell's ability to metabolize ROS. In addition, LOX activities which are responsible for lipid peroxidation are downregulated in NaHS-treated aleurone layers at early stage of treatment (Figures 3(e) and 5(e)). Meanwhile, quantitative-PCR analysis shows that expression of* HvSOD1*,* HvAPX*,* HvCAT1,* and* HvCAT2* genes in non-GA-treated layers is maintained at higher levels in NaHS treatment compared with water controls (Figure 4). Consistent with lower LOX activity in NaHS-treated aleurone layers, the accumulation of LOX transcripts is also reduced (Figure 4). In summary, H_2_S slows down ROS-induced PCD in barley aleurone layers probably by enhancing the activity and expression of ROS-scavenging enzymes and reducing the peroxidation of membrane lipids.

Consistently, Xie et al. [18] found that H_2_S delayed GA-triggered PCD in wheat aleurone layers by increasing GSH content and heme oxygenase-1 gene expression. Here we provide evidence that H_2_S can alleviate both natural PCD and GA-triggered PCD through the modulation of antioxidant enzyme activities and their expression. Compared with the slow natural PCD process, the present study also confirms the pivotal role of GA in triggering PCD (Figure 5).

The role of ROS in GA and ABA signaling in barley aleurone cells is recently clarified [30], in which they found that the production of H_2_O_2_, a type of ROS, was induced by GA in aleurone cells but suppressed by ABA. Furthermore, exogenous H_2_O_2_ appeared to promote the induction of *α*-amylases by GA by promoting the expression of* GAMyb* and *α*-amylase genes, whereas antioxidants suppressed the induction of *α*-amylase. Unexpectedly, we found that H_2_S reduces ROS accumulation and delays PCD process in barley aleurone layers in the presence or absence of GA and meanwhile promotes the secretion of *α*-amylase, suggesting that the antioxidant H_2_S works through an unknown way to regulate *α*-amylase secretion and antioxidants do not always suppress the induction of *α*-amylase. Besides, the activation of *α*-amylase by H_2_S in the absence of GA implies that *α*-amylase can be secreted independent of GA signaling pathway. Therefore, the present findings advance our knowledge on the relations between PCD process and *α*-amylase secretion and the independence of *α*-amylase secretion and GA pathway.

The activation of cysteine proteases was instrumental in the PCD of soybean cells, while cystatin, an endogenous cysteine protease inhibitor gene, inhibited cysteine protease activity and blocked PCD in these cells [7]. In this paper, we show that H_2_S downregulates the transcriptions of two barley cysteine proteinases,* HvEPA* and* HvCP3-31,* in non-GA-treated barley aleurone layers, thereby delaying cell component degradation and PCD process.

## 5. Conclusion

In summary, we report the role of H_2_S in delaying PCD in barley aleurone layers regardless of the presence or absence of GA without repressing *α*-amylase induction, suggesting that the function of H_2_S may be universal in regulating plant PCD. PCD in plant cells is regulated by many internal and external factors, such as the hormones (GA and ABA), Ca^2+^, ROS, and NO [6]. It will be interesting to know whether H_2_S is involved in other signals and how *α*-amylase is induced by H_2_S in the presence or absence of GA in cereal aleurone cells.
